# Systemic delivery of human GlyR IgG antibody induces GlyR internalization into motor neurons of brainstem and spinal cord with motor dysfunction in mice

**DOI:** 10.1111/nan.12666

**Published:** 2020-09-28

**Authors:** A. Carvajal‐González, L. Jacobson, L. Clover, M. Wickremaratchi, S. Shields, B. Lang, A. Vincent

**Affiliations:** ^1^ Nuffield Department of Clinical Neurosciences John Radcliffe Hospital Oxford UK; ^2^ Hurstwood Park Neurological Centre Brighton and Sussex University Hospitals NHS Trust West Sussex UK; ^3^ Neurosciences Department Taunton and Somerset NS Foundation Trust Musgrove Park Hospital Taunton UK; ^4^ Present address: School of Medicine Dr A Carvajal‐Gonzalez Universidad El Bosque Bogotá Colombia

**Keywords:** animal model, antibody‐mediated autoimmune disease, glycine receptor, PERM, Progressive encephalomyelitis with rigidity and myoclonus, stiff person syndrome

## Abstract

**Aims:**

Progressive encephalomyelitis with rigidity and myoclonus (PERM) is a life‐threatening condition often associated with highly raised serum antibodies to glycine receptors (GlyRs); these bind to the surface of large neurons and interneurons in rodent brain and spinal cord sections and, *in vitro*, inhibit function and reduce surface expression of the GlyRs. The effects *in vivo* have not been reported.

**Methods:**

Purified plasma IgG from a GlyR antibody‐positive patient with PERM, and a healthy control (HC), was injected daily into the peritoneal cavity of mice for 12 days; lipopolysaccharide (LPS) to open the blood–brain barrier, was injected on days 3 and 8. Based on preliminary data, behavioural tests were only performed 48 h post‐LPS on days 5–7 and 10–12.

**Results:**

The GlyR IgG injected mice showed impaired ability on the rotarod from days 5 to 10 but this normalized by day 12. There were no other behavioural differences but, at termination (d13), the GlyR IgG‐injected mice had IgG deposits on the neurons that express GlyRs in the brainstem and spinal cord. The IgG was not only on the surface but also inside these large GlyR expressing neurons, which continued to express surface GlyR.

**Conclusions:**

Despite the partial clinical phenotype, not uncommon in passive transfer studies, the results suggest that the antibodies had accessed the GlyRs in relevant brain regions, led to antibody‐mediated internalization and increased GlyR synthesis, compatible with the temporary loss of function.

## Graphical abstract

Purified IgG from a patient with progressive encephalopathy with rigidity and myoclonus (PERM) who had raised antibodies to the inhibitory glycine receptor was injected into mice together with lipopolysaccharide to open the blood–brain barrier. The mice developed a motor phenotype, indicated by falling off a rotating cylinder earlier than healthy IgG injected controls. At termination, these mice had deposits of IgG within the brainstem neurons which express glycine receptors, consistent with an antibody‐induced dysfunction of these neurons.

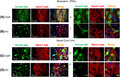

## Introduction

There is growing evidence that antibodies to receptors, ion channels or related proteins are important biomarkers for a range of neurological and neuropsychiatric diseases that often improve with immunotherapies [1]. Many of these antibodies cause loss or inhibition of synaptic proteins resulting in changes in neuronal activity [1, 2]. Progressive encephalomyelitis with rigidity and myoclonus (PERM) is a rare neurological disease that can be very severe and potentially fatal [3]. It is characterized by muscular rigidity, stimulus‐sensitive spasms, myoclonus, hyperekplexia, brainstem dysfunction, autonomic dysfunction and variable sensory symptoms. In 2008, antibodies to the glycine receptor (GlyR) were identified retrospectively in a patient with PERM who eventually improved substantially following intensive immunotherapies [4]. From then, many patients with GlyR antibodies have been reported as case series [5, 6, 7, 8, 9]. Although PERM exhibits many overlapping symptoms with stiff person syndrome (SPS), PERM with GlyR‐Abs is now considered a distinct antibody‐mediated syndrome with better treatment response [1, 2, 5].

The GlyR is a ligand‐gated ion channel that mediates inhibitory neurotransmission in the central nervous system (CNS) [10]. It is predominantly expressed on the surface of motor neurons and also on the inhibitory neurons in the spinal cord and brainstem, regions involved in motor regulation [11, 12]. Loss of glycinergic inter‐neuronal inhibition in these regions causes enhanced excitability of motor neurons that could lead to the stiffness and spasms seen in PERM [13, 14]. For example, in transgenic mice expressing a dominant mutation of the GlyRα1 subunit, disruption of glycinergic neurotransmission causes motor symptoms similar to those observed in patients with PERM [15] and alters the pattern of alternating spinal cord rhythms [16]. We previously showed, by indirect immunohistology, that GlyR‐Abs bind to rodent spinal cord and brainstem colocalizing with monoclonal antibodies to glycine receptor‐alpha1. *In vitro*, after incubation at 37°C, the patient IgG antibodies caused internalization and lysosomal degradation of GlyRs on GlyR‐transfected human embryonic kidney (HEK) cells [5], consistent with an antibody‐mediated pathogenesis. GlyR antibodies also inhibited GlyR currents in cultured spinal cord motor neurons [17] indicating that the antibodies can reduce GlyR function.

Despite these compelling *in vitro* results, passive transfer of GlyR‐specific antibodies into experimental animals, the main criteria for defining autoantibody‐mediated disease [18], has not been reported. Moreover, although antibody titres are often much higher in serum than CSF [1, 5], most transfers of neuronal and glial antibodies have involved injection or infusion of purified IgG or CSF into the cerebral parenchyma, cerebral ventricles or spinal cord. Studies of systemic injection of antibodies are rare and have required some additional insult to breach the blood–brain barrier (for a recent review, see [19]). Here we use intraperitoneal injection and LPS to look at the *in vivo* effects of GlyR antibodies.

## Material and Methods

### *In vitro* investigations

#### Sera and patient plasma

Sera were obtained from the Oxford Neuroimmunology service that routinely receives samples for antibody screening by a cell‐based assay expressing the GlyR alpha subunit as antigen [4, 5]. Plasmapheresis material was available from a patient with PERM with raised GlyR‐Abs and control plasma from a 72‐year‐old healthy male donor (GlyR‐antibody negative).

#### Ethics

Use of the patient sera for research was approved by the Oxford Regional Ethics Committee Ref: 07/Q1604/28. The patient and control consented separately for use of their plasma in experimental studies.

#### GlyR antibody detection

HEK 293T cells were transfected with GlyRα1 subunits for serum and plasma antibody testing. The titres of the patients’ sera and IgG preparations were determined by identifying the dilution at which the binding was scored as 1 (on scale from 0 to 4; as in [5, 20]).

#### IgG purification

For *in vivo* experiments IgG fractions were purified from the plasmapheresis filtrates of the patient, obtained as part of standard clinical care, and from the healthy control plasma. Ammonium sulphate precipitation was used to partially purify and concentrate the large amounts of IgG required [21]. The IgG preparations were dialysed, concentrated and sterile filtered and their concentrations determined by western blotting. Details are available in Data S1.

#### Effects of antibodies on GlyR expression

For examining the effects of serum GlyR antibodies on surface expression of EGFP‐GlyRs in HEK cells, sera scoring 3 (at 1:80–1:160 dilutions) were added for 1 h at 4ºC and then removed by brief washing. The live cells were then incubated at 37ºC for 5 min to 2 h, and surface EGFP‐GlyRs were measured after fixation. Bound human IgG was detected with Alexa‐fluor goat anti‐human IgG 568 (red; 1:750, Invitrogen). To prevent new GlyR synthesis, cycloheximide (50 µg/mL, Sigma‐Aldrich, Dorset, UK) was added to the growth medium for 1 h before the addition of the sera. The mean ± SEM of three experiments were plotted.

### In vivo experiments

#### Mice

All C57/BL6j male mice (6–8 weeks old) were purchased by the Biomedical Service from Charles River, Kent, UK Ltd. Animals were kept with *ad libitum* access to food and water; 12:12 light:dark cycle with lights on at 7 am. After habituation, they were randomized to the different groups. Animal use and care were in accordance with the United Kingdom Home Office Animals in Scientific Procedures Act (1986) and with institutional guidelines under HO PPL 40/3581. All samples were coded, and experiments and analyses conducted blind to case status. The protocols are shown in Figure S1.

#### Time course of effect of intraperitoneal injection of LPS on mouse behaviour

LPS causes physiological and behavioural alterations (sickness behaviour [22]) due to the systemic release of pro‐inflammatory cytokines. To see the duration of such effects, measures of general health, temperature, motor behaviour, anxiety‐like behaviour and weight were measured immediately after the injection of LPS and subsequently over 48 h. The protocol and results are described in Figures S1 and S2.

#### Effects of injection of IgG or saline with LPS comparing with untreated mice

Purified IgG from the GlyR‐antibody PERM patient (GlyRα1‐Ab IgG; 12.4 mg/mL) and one healthy control (HC IgG; 14.6 mg/mL) was used. Twenty‐four C57/BL6j male mice (6–8 weeks old) were randomized (*n* = 6 for each group) to receive intraperitoneal (i.p.) injections of 0.5 mL of each IgG preparation, or saline only, over 12 consecutive days. LPS was given on days 3 and 8 at 3 mg/kg and 1.5 mg/kg respectively. A fourth group was left untreated throughout.

### Behavioural tests

Observation and behavioural tests (see Data S1) were aimed mainly at identifying mouse behaviours that could be related to the motor manifestations observed in PERM patients and were conducted with the investigator blinded to the treatment group. The mice were watched for any obvious stiffness or spasms during the behavioural testing [23]. The rotarod, grip strength, beam walking and footprint analyses were used for motor functions, [24] and the light–dark box and open field were used for anxiety‐like responses [25]. Grip strength was tested with the Kondziela's inverted screen [26]. To analyse gait, footprint analysis was used on a wide runaway. Postural tone and sensorimotor function were analysed with a skilled walking test along the narrow beam [27] with slight modifications [28, 29]. All behaviours were recorded with a video camera (NV‐GS27, Panasonic) and the results analysed, still coded, offline after the experiments were finished.

### Immunohistochemical analysis of mouse brains *ex vivo*

At termination on day 13, mice were deeply anaesthetized with intraperitoneal injections of ketamine (100 mg/kg) and xylazine (10 mg/kg) and, after withdrawing blood from the heart, perfused with PBS (see Data S1); the brain and spinal cord was removed and snap‐frozen and embedded. Eleven‐micron thick cryostat (Leica 1850 CM, Germany) sagittal fresh frozen sections were thaw‐mounted on SuperFrost Plus glass slides, moistened with PBS and fixed with formaldehyde 3% for 5 min. The sections were blocked at room temperature (RT) for 1 h in 10% normal goat serum before use. To determine human IgG deposition, brain and spinal cord sections were incubated at 4°C overnight with a polyclonal CF633 goat anti‐human IgG (1:1000; Biotium, CA, USA); this had been preabsorbed against mouse IgG to avoid cross‐reaction with mouse IgG present in the brain tissue. To demonstrate the localization of the human IgG deposits, sections were incubated with CF633 goat anti‐human IgG and with primary mouse monoclonal antibody to Von‐Willebrand factor (1:2000; Dako, Eindhoven, Netherlands) as above, washed and incubated with Alexa Flour 568 anti‐mouse IgG (1:1000; Invitrogen, UK) for 1 h at RT, coupled with DAPI counterstaining. The anti‐mouse IgG had been cross‐adsorbed against bovine, goat, rabbit, rat and human IgG and human serum. The number of IgG deposits on coded sections was counted by two independent observers.

To demonstrate the binding specificity of the human IgG deposits and characterize the neurons with human IgG deposits, sections were incubated with CF633 goat anti‐human IgG and with primary rabbit polyclonal antibody to GlyRα1 (GlyR‐polyAb2; 1:500; Synaptic Systems, Göttingen, Germany) as above, washed and incubated with Alexa‐Fluor 568 donkey anti‐rabbit IgG (1:1000, Invitrogen, UK) for 1h at RTand coupled with DAPI counterstaining. The coverslips were covered with mounting medium (Dako) and images were photographed under a Leica fluorescence microscope (DM 2500) with the appropriate filter settings with a digital camera (QImaging, Rolera XR, Fast 1394) or with a Zeiss confocal microscope (LSM 710).

### Statistical analysis

All statistical analyses were performed using SPSS version 18 for windows (SPSS Inc., Chicago, USA) and all figures were created using GraphPad Prism version 7 for windows (GraphPad Software, San Diego, California, USA). The data were presented as mean ± standard error for results from individual mice at each time point. Variables were tested for differences using Student´s *t*‐test, One‐way ANOVA, Two‐way ANOVA or Kruskal–Wallis one‐way analysis of variance depending on the number of groups and the data distribution were used.

## Results

### In vitro experiments

#### GlyR‐IgG‐mediated GlyR loss from the surface of transfected cells is restored by insertion of new GlyRs

**Figure 1:**
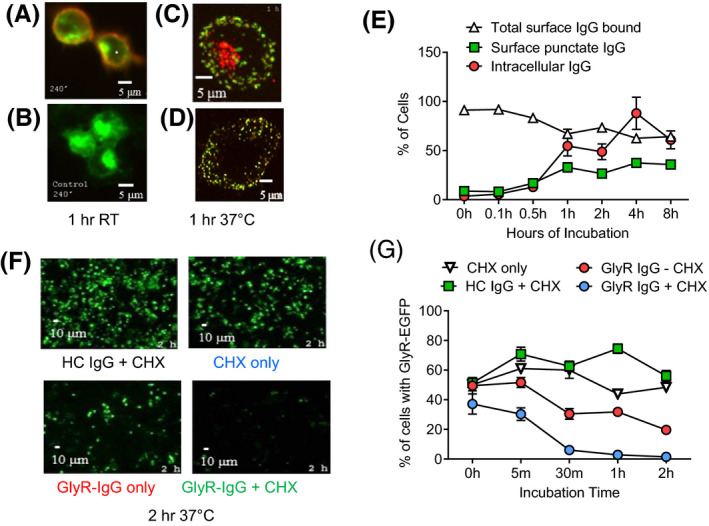
GlyR‐EGFP internalization caused by GlyR‐Ab is partially compensated by ongoing GlyR expression (**a**) GlyR‐Abs (red) bind to GlyR‐EGFP (green) HEK cells at 1 h room temperature compared with control IgG (**b**). At 37°C, GlyR‐Abs are internalized as detected after permeabilization (**c**) but are still present on the surface where IgG binds in a punctate pattern (**d**). Mean results from two sera showing increase of internal IgG and surface punctate IgG (Two‐sided Student’s *t*‐test *t* = 6308 df =8, *P* = 0.0002) over time with a slight decrease in total IgG bound (**e**) (Two‐sided Student’s *t*‐test *t* = 6308 df = 8, *P* = 0.0002). Incubation of GlyR‐EGFP (green) HEK cells with GlyR‐IgG over 2 h leads to a loss of EGFP‐GlyR compared to incubation with HC‐IgG (**f**). Results from two experiments from one sera shows a decrease of the proportion of GlyR‐EGFP cells when incubated with GlyR‐IgG at 37°C compared to incubation with HC‐IgG; the effect is even greater with cycloheximide treatment that prevents new GlyR synthesis (**f, g**) (Two‐way repeated measures ANOVA *F* (12, 80) = 8,883 *P* < 0.0001).

As previously reported [5], IgG antibodies bind to glycine receptors on the surface of GlyR‐transfected HEK293 cells (e.g. **Figure** **1a**), whereas control IgG does not bind (**Figure** **1b**). After incubation at 37°C, these antibodies caused internalization of the glycine receptor; GlyR‐IgG (detected with green fluorescent anti‐IgG) initially bound in a diffuse distribution to GlyRs, but after 1 h showed a punctate pattern, suggesting divalent IgG binding had caused GlyR aggregation. After fixation and permeabilization, intracellular IgG was detected by a red secondary anti‐human IgG (e.g. **Figure** **1c**). Nevertheless, despite the internalization of GlyRs, those on the surface were not substantially reduced (**Figure** **1d**). The changes over time are shown in **Figure** **1e**. The total surface IgG bound to GlyRs decreased over the incubation period of 2 h by about 30% (two‐sided Student’s *t*‐test *P* = 0.0002), but even as the number of cells with intracellular IgG increased, so did the number of cells with IgG puncta on their surface (two‐sided Student’s *t*‐test *P* = 0.0002).

This suggested that GlyR synthesis (which would be ongoing in the transfected cells) could restore the surface expression, even in the continued internalization by the GlyR‐Abs. To demonstrate this more clearly, we performed a time course of GlyR‐EGFP expression, using cycloheximide to inhibit the synthesis of new GlyR‐EGFP, and looked at the proportion of cells expressing GlyR‐EGFP after incubation with GlyR‐IgG and HC‐IgG. Examples are shown in **Figure** **1f**. Fifty‐six per cent of HC‐IgG‐treated cells showed GlyR‐EGFP expression in the total cells (identified by DAPI staining; not shown) after 2 h of incubation with minimal decrease even in the presence of cycloheximide. When incubated with GlyR‐IgG, by contrast, the cells expressing GlyR‐EGFP fell from 49.45% at 0 h to 19.63% after 2 h (**Figure** **1g**; two‐way repeated measures ANOVA *P* < 0.0001 in time). However, in the presence of cycloheximide, the percentage of cells expressing GlyR‐EGFP fell from 37.1% at 0 h to 1.43% at 2 h in the GlyR‐IgG (**Figure** **1g**; two‐way repeated measures ANOVA *P* < 0.0001 in condition, time and interaction). These results confirm that new synthesis of GlyRs can replace those that are lost, at least in transfected HEK cells, but whether this happens *in vivo* is not yet known.

### *In vivo* experiments: results of passive transfer of GlyR‐antibodies

#### PERM patient GlyR‐IgG affected forced walking on the rotarod

The patient was a 40‐year‐old man with known PERM [30]; a summary of his clinical details is presented in Table 1. Three LPS‐treated groups (GlyR‐Ab, HC and saline) were compared with un‐injected mice. Based on the results of the LPS experiment (see Figure S2), behavioural testing was performed starting 48 h after each LPS injection for 3 days on days 5–7 and 10–12. The protocol is shown in Figure S1.

**Table 1:** Clinical details of 40‐year‐old male with GlyR‐antibody PERM

Presentation	Progression	Other clinical aspects	Investigations	Treatment and outcome
Subacute onset of respiratory difficulties, involuntary jerking and dysphagia after a 5‐ day prodromal urinary tract infection	Respiratory difficulties required ventilation. Marked ophthalmoplegia and rigidity with stimulus induced myoclonic jerks to noise and touch	Awake, appropriate behaviour and no cognitive impairment.	CSF: 11 monocytes, OCB negative, normal glucose protein, virology and cultures negative.	Intravenous immunoglobulins, then oral steroids. Required oral and intrathecal baclofen
		Benign anogenital papillomatous lesions	EMG: continuous motor unit activity	At time of first study [5] he was still ventilator dependent. Improvement continued, he suffered one relapse, but eventually good outcome [30].
			Max GlyR‐Ab: Serum: 1:600, CSF: 1:40	
			Brain CT scan and MRI normal.	

CSF, Cerebral spinal fluid; OCB, Oligoclonal bands, EMG, Electromyography, CT, Computerized tomography scan, MRI, Magnetic resonance imaging. Data are from unpublished information and [5, 30].

**Figure 2:**
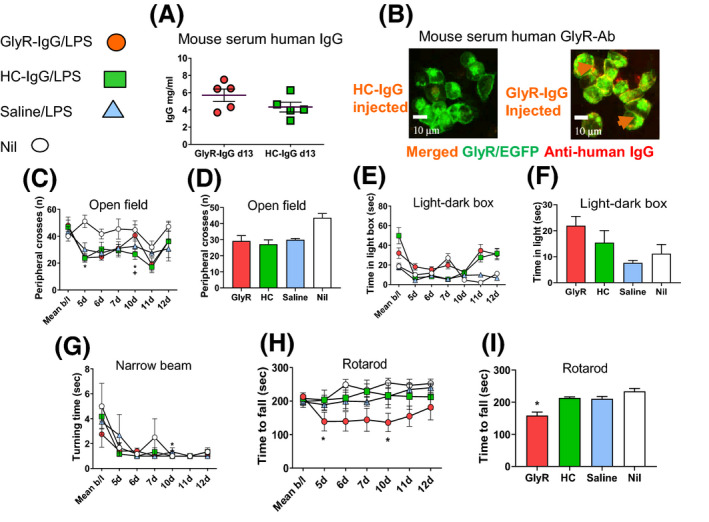
Passive transfer of GlyR‐IgG from a PERM patient affects forced walking ability. Behavioural testing of adult mice exposed to GlyR‐IgG, HC‐IgG or saline. (**a**) On day 14 when the behavioural testing had been finished, human IgG was found in both GlyR‐IgG and HC‐IgG‐injected mice with no difference between them (Two‐sided Student’s *t*‐test *t* = 1486 df = 8, *P* = 0.176). (**b**) Cell‐based assay showed that GlyR‐Abs were present in the GlyR‐IgG mice although not at high titre (arrows (orange) point to the merged GlyR‐Ab (red) bound to the surface of GlyR‐EGFP cells (green)). (**c–g**) Examples of some test results are shown. (**c, d**) In the open field, all three LPS groups showed a decrease in peripheral crosses compared with the non‐injected mice (One‐way ANOVA *F* = 9.810 df = 3 *P* < 0.0001). (**e, f**) In the dark/light boxes there was a suggestion of increased time in the light in the dark/light box in the GlyR‐IgG group but no significant difference from HC‐IgG‐injected mice. (**g**) The GlyR‐IgG mice took more time to turn around on the narrow beam before traversing the beam at baseline, but the four groups did not differ thereafter. (**h, i**) Mice injected with GlyR‐Ab i.p. showed a significant decrease in the time before falling off the rotarod compared to mice treated with HC IgG, saline or untreated mice. The effect was most clearly evident on day 10, and when compared to baselines the GlyR‐IgG‐treated mice spent significantly less time on the rotarod at days 5 and 10 of injection (*); (**h**) Two‐way ANOVA *F* = 17.24 df = 162 *P* < 0.0001 and (**i**) Summary of the data during the injected days One‐way ANOVA (*F* = 30.0 df = 23 *P* < 0.0001).

Bleeding the mice at termination (13 days) showed human IgG in both GlyR‐Ab‐ and HC IgG‐treated groups (5.7 mg/mL and 4.3 mg/mL respectively, two‐sided Student’s *t*‐test *P* = 0.176, **Figure** **2a**), but GlyR‐Ab was detected only in the GlyR‐IgG‐treated mice (**Figure** **2b**). There was no evidence of spasms or rigidity in the mice in their cages. The behavioural results are presented as daily tests, comparing with baseline results, and also as means of the four groups during the injections. On the open field test, all three LPS groups had reduced activity compared to the un‐injected controls (one way ANOVA *P* < 0.0001; **Figure** **2c,d**) but results were variable within each group and there was no differences in the mean values for time spent in the light box (**Figure** **2e,f**) or in the time to turn around on the narrow beam (**Figure** **2g**). However, the GlyR‐IgG mice struggled on the accelerating rotarod and fell off earlier than the other groups (**Figure** **2h,i**, two‐way ANOVA *P* < 0.0001 in condition, time and interaction). The data at each time point shows that this was consistent throughout days 5–10 (significant at days 5 and 10) but had reversed by days 11 and 12. Detailed analyses of the behavioural results are shown in Table 2.

**Table 2:** Results of the behavioural effects of PERM patient GlyR IgG

Test	GlyR IgG Mean ± SE. *n* (6)	HC IgG Mean ± SE. *n* (6)	Saline Mean ± SE. *n* (6)	Non‐injected Mean ± SE. *n* (6)	*P* value* ANOVA Column factor	*P* value* GlyR v. HC Post hoc
Weight (g)	24.44 ± 0.37	23.96 ± 0.56	24.39 ± 0.60	27.30 ± 0.51	*F* (3, 134) = 56.14; *P* < 0.0001	*P* = 0.70
Open field, peripheral crosses (*n*)	32.33 ± 5.90	30.00 ± 4.71	31.82 ± 4.04	43.11 ± 4.87	*F* (3, 134) = 9.11; *P* < 0.0001	*P* = 0.91
Open field, total crosses (*n*)	29.86 ± 6.19	32.21 ± 5.43	33.26 ± 4.35	47.87 ± 5.67	*F* (3, 134) = 11.07; *P* < 0.0001	*P* = 0.92
Inverted mesh strength test, time (s)	266.66 ± 11.36	252.11 ± 17.37	261.79 ± 15.84	258.93 ± 22.23	*F* (3, 134) = 0.79; *P* = 0.50	*P* = 0.68
Wide runaway, time (s)	13.49 ± 1.21	13.75 ± 1.66	13.39 ± 1.67	14.10 ± 1.71	*F* (3, 134) = 0.25; *P* = 0.86	*P* = 0.99
Wide runaway, number of steps	19.36 ± 0.69	19.82 ± 0.73	18.85 ± 0.79	19.15 ± 0.72	*F* (3, 134) = 1.51; *P* = 0.22	*P* = 0.78
Light–dark compartment, crosses (*n*)	3.40 ± 0.77	2.74 ± 0.59	2.50 ± 0.48	2.60 ± 0.40	*F* (3, 134) = 2.01; *P* = 0.12	*P* = 0.51
Light–dark compartment, time in dark, (s)	279.30 ± 9.55	285.31 ± 8.30	286.89 ± 6.13	287.50 ± 5.00	*F* (3, 134) = 2.23; *P* = 0.09	*P* = 0.36
Light–dark compartment, time in light, (s)	20.70 ± 9.55	14.69 ± 8.30	13.11 ± 6.13	12.50 ± 5.00	*F* (3, 134) = 2.23; *P* = 0.09	*P* = 0.36
Successive alleys, time in closed alley (s)	290.49 ± 2.75	293.38 ± 1.96	290.73 ± 3.42	288.75 ± 3.49	*F* (3, 134) = 2.25; *P* = 0.09	*P* = 0.46
Successive alleys, time in open alleys time (s)	9.10 ± 2.48	6.46 ± 1.97	8.89 ± 3.33	11.23 ± 3.21	Kruskal–Wallis: H (4) = 5.45; *P* = 0.14	Dunn’s *P* > 0.99
Narrow Beam, time to turn (s)	1.36 ± 0.21	1.57 ± 0.24	1.68 ± 0.44	1.95 ± 0.61	*F* (3, 134) = 0.96; *P* = 0.41	*P* = 0.99
Narrow beam, total time to traverse (s)	8.44 ± 0.79	9.93 ± 1.28	9.67 ± 1.41	10.18 ± 1.67	*F* (3, 134) = 2.05; *P* = 0.11	*P* = 0.27
Narrow beam, number of steps	18.88 ± 0.57	19.48 ± 0.76	18.92 ± 0.60	18.65 ± 0.65	*F* (3, 134) = 1.72; *P* = 0.17	*P* = 0.46
Narrow beam, walking score	7.13 ± 0.35	7.01 ± 0.50	7.20 ± 0.35	7.55 ± 0.26	*F* (3, 134) = 2,24; *P* = 0.09	*P* = 0.95
Rotarod, time (s)	149.45 ± 31.29	214.04 ± 34.75	212.51 ± 18.53	239.51 ± 15.82	*F* (3, 134) = 17.39; *P* < 0.0001	*P* = 0.0005

**SE,** Standard error**, *n*,** Number of mice, **g,** Grams, **s,** Seconds. *Two‐way ANOVA, Column factor (group), row factor (day), Post hoc analysis **Tukey’s** multiple comparison. Non‐significant if *P* > 0.05.

#### IgG deposited in brain regions known to express glycine receptors

**Figure 3:**
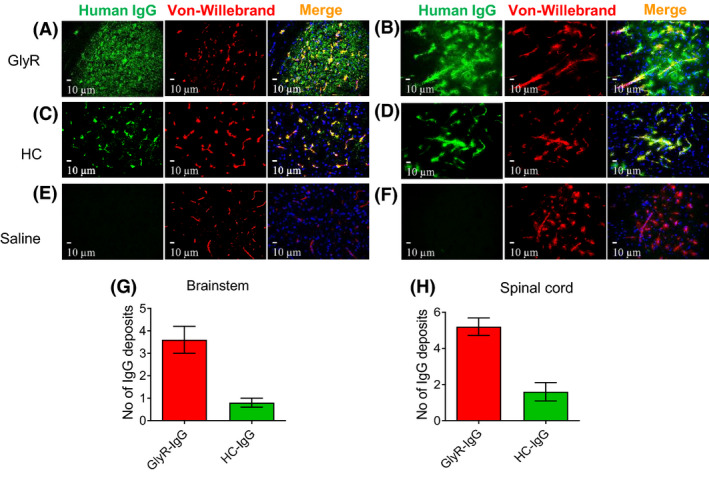
IgG deposits were found in different brain regions involved in motor control particularly the brainstem and spinal cord. Double labelled photographs were taken at 40× magnification at the brainstem in the PnC and the ventral horn of the spinal cord. In GlyR‐IgG‐treated animals, human IgG (green) is located inside the vessels colocalizing with Von‐Willebrand factor antibody (red), and also in patches within these brain regions without any colocalization (**a, b**). In healthy control‐treated animals, the human IgG (green) is located only in the vessels (**c, d**). In saline‐treated animals (**e, f**) and uninjected controls (not shown), human IgG was not detectable. (**g, h**) Quantitative analysis shows the mean number of patches of IgG seen in the two IgG‐injected groups, in the brainstem (Two‐sided student *t*‐tests: *t* = 4.427 df = 8, *P* = 0.002) (**g**) and in the spinal cord (*t* = 5.091 df = 8, *P* < 0.001) **(h**). Nuclei are stained with DAPI (blue).

After termination, the mouse brains were perfused and fixed for immunofluorescence. Human IgG deposits could be found in many brain regions including the brainstem (midbrain, pons and medulla oblongata), the spinal cord (ventral and dorsal horns), the hippocampus (CA regions and dentate gyrus) and the cerebellum (white matter, granular and molecular layer) in both the GlyR IgG‐treated mice and the HC IgG‐treated mice. Results of the brainstem and spinal cord are shown in Figure 3 and of cerebellum and hippocampus in Figure S3. As expected, much of the IgG was deposited within vessels, colocalizing with Von‐Willebrand immuno‐reactivity in both IgG‐injected groups (e.g. **Figure** **3a–d**; Figure S3); in the saline‐injected (and untreated, data not shown) group, only the Von‐Willebrand reactivity was observed (**Figure** **3e,f**). Strikingly, in addition, there was patchy diffuse distribution of IgG around the vessels and in the neuropil (**Figure** **3a,b**; Figure S3), and on scanning at low magnification, these were substantially more frequent in the GlyR‐IgG‐injected mice than in the HC‐injected mice (**Figure** **3g,h**; two‐sided Student’s *t*‐test *P* = 0.002; *P* = 0.0009 respectively).

#### IgG deposits were localized intracellularly as well as in the neuropil

**Figure 4:**
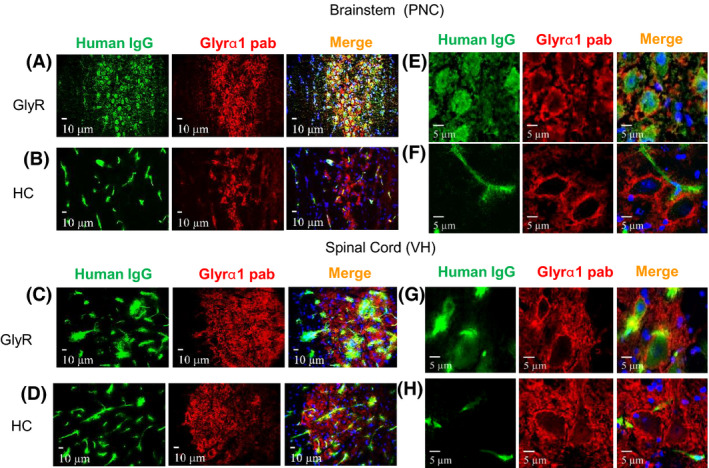
IgG deposits were found in brain regions that express GlyRα1. Double labelled images from the PnC of the brainstem and ventral horn of the spinal cord. In GlyR‐IgG‐treated animals, human IgG (green) colocalizes with GlyRα1 antibody (red), and on close inspection some IgG is within the cytoplasm (asterisks (**a, c**)). In healthy control‐treated animals, the human IgG (green) is located only in the vessels and does not colocalize with GlyR (**b, d**). (**e, g**) Higher resolution images confirm that IgG deposits are localized intracellularly in both brainstem and spinal cord. In addition, there is human IgG bound on the surface, apparently separated from GlyRα1 (red). In HC‐IgG‐treated animals only vascular human IgG (green) is seen (**f, h**). Nuclei are stained with DAPI (blue). Confocal photographs were taken at 63× magnification and slightly cropped images are shown.

The brains were also imaged to compare the human IgG binding with GlyRα1 (identified with the GlyR polyAb2). In the cerebellum there was some colocalization with GlyRs, but in the hippocampus, where GlyRα1 is very scarce, there was no colocalization (Figure S3). The results of the brainstem and spinal cord were more informative. Human IgG colocalized with GlyRa1 in the PnC and in the ventral horns of the spinal cord (**Figure** **4a,c**) in the GlyR‐IgG‐injected but not in the HC‐injected mice (**Figure** **4b,d**). Moreover, confocal images (**Figure** **4e,g**) showed that GlyRα1 immunoreactivity was not only in the neuropil and in aggregates on the surface of the large neurons, but was also clearly intracellular within those neurons, in complete contrast to the results in the HC‐injected mice (**Figure** **4f,h**). Importantly, despite the IgG bound to the surface and within the neurons, consistent with IgG‐mediated GlyR internalization, there was also membrane GlyR on the surface of these neurons as detected by GlyR polyAb2.

## Discussion

Glycine receptor antibodies have been identified in patients with a range of symptoms including muscle spasms, hyperekplexia, stiffness and rigidity, autonomic, cerebellar and brain stem dysfunction, often referred to as PERM. Although these defects are ascribable to loss of GlyRs from the glycinergic neurons in the relevant CNS regions, particularly the brainstem and spinal cord, the *in vivo* effects of GlyR antibodies have not previously been explored. Here we transferred purified serum IgG antibodies into mice over 12 days giving them two injections of LPS in order to make the CNS more vulnerable. Although the clinical phenotype from the IgG transfer was not substantial, there was a motor deficit seen with PERM patient IgG that could be attributed to GlyR dysfunction. However, this deficit appeared to reverse over the time of the injections, hinting at some compensatory mechanism operating in the target tissues. Indeed, *post mortem* studies not only showed IgG deposition in brainstem, cerebellum and spinal cord, compared with minimal deposits in the hippocampi and cortex, but also showed intracellular deposition of the human IgG despite ongoing surface expression of GlyRs. Thus, both *in vitro* experiments (Figure 1) and detailed *post mortem* confocal microscopy (Figure 4) suggest that there is GlyR‐IgG induced internalization of GlyRs that is accompanied by compensatory increases in surface expression of GlyRs.

The blood–brain barrier was artificially disrupted to allow access of GlyR‐Abs to the brain. There are very few studies of passive transfer of antibodies mediating CNS diseases via systemic injection, and most of the examples are for neuromyelitis optica [31, 32], stiff person syndrome [33] and paediatric autoimmune neuropsychiatric disorders associated with streptococcal infection [34]. Only recently have CASPR2 antibodies been injected systemically into mice in experiments similar to those reported here [35]. In fact, LPS injection into rodents induces behavioural changes, called sickness behaviour, secondary to the neural effects of cytokines [22, 36, 37], and a range of abnormal behaviours and physiological measures were found during the first 24–48 h after LPS injection (Figure S2). It is important to take the duration of these changes into account when using LPS in experimental animals.

Passive transfer of GlyR‐Abs produced a pattern of motor dysfunction characterized by impaired forced walking ability, but other typical PERM manifestations such as stiffness, exaggerated startle response or muscle spasms were not seen. The motor dysfunction observed in the mice was likely caused by a local action of the injected IgG on supra‐spinal and spinal motor pathways, as IgG deposits were observed in brain regions that express glycine receptors and are involved in control of motor function [38]. The lack of clear anxiety‐like symptoms, as evidenced by normal behaviour in the light/dark compartment test, might have been expected as this symptom is not frequent in PERM patients [5]. In fact, the GlyR‐IgG‐injected mice spent a little more time in the light (Figure 2, Table 2), consistent with an anxiolytic effect. Interestingly, anxiety is common in stiff person syndrome with GAD antibodies [3], and in a parallel experiment of IgG from an SPS patient, the mice spent less time in the light (AC, AV unpublished results).

Our previous experiments showed that GlyRs were internalized in transfected HEK cells where they colocalized with LAMP2 in lysosomes; the results also hinted at a replacement of surface GlyRs [5] as shown more clearly here. It was striking that after 12 days of GlyR IgG injections there was clear evidence of IgG within the large motor neurons of the brainstem PnC and spinal cord ventral horns, consistent with internalization. Moreover, there was also GlyRs still available on the surface of the same neurons. Together these observations strongly suggest that binding to the GlyRs and internalization is, at least over the 12 days of these experiments, associated with replacement by newly synthesized GlyRs. One can speculate that this apparent replacement could be responsible for the relatively modest phenotype that we observed in the mice, and the return of the rotarod activity to control levels by the time of the final test. On the other hand, recent studies on mouse motor neurons *in vitro* showed that four purified GlyR‐Ab IgG preparations directly blocked GlyR currents [17]. It appears that, unusually for neuronal antibodies [2], GlyR antibodies can cause direct block of function as well as internalization. The final effects of the antibodies *in vivo* are likely to be a balance between internalization, complement‐dependent neuronal loss, compensatory changes and direct inhibition.

There are a number of limitations in this work. Although it was not possible to reproduce the clinical core features of PERM patients, this is not uncommon in passive transfer studies where most systemic studies have failed to model all or any of the core features of the human disease in animals as discussed above, except for one study of an SPS patient where there was short‐lived stiffness in rats, associated subsequently with convincing evidence of neuronal dysfunction *ex vivo* [33, 39]. Similar difficulties in obtaining a full clinical phenotype are found with intraventricular injection of antibodies. For example, infusion of NMDA receptor CSF antibodies into mice, with clear binding to the hippocampi, did not produce any evidence of movement disorder, one of the key features of the human disease [40]; a single injection of NMDAR IgG [41] or infusion of LGI1 antibodies [42], both of which are associated with epilepsy, produced no spontaneous seizures although there was EEG or *ex vivo* evidence of neuronal hyperexcitability.

Further studies could transfer more IgG, from patients with a higher titre of antibodies, specific human monoclonal antibodies, or use chronic infusion of IgG into the ventricles. GlyR antibody serum levels can be very high with variable intrathecal synthesis [5], and a comparison between the peripheral and central routes of passive transfer might prove interesting. As supra‐spinal disinhibition has been demonstrated in SPS with GAD autoimmunity [43], *in vitro* electrophysiological studies of glycinergic neurotransmission in slices of the brainstem and ventral horn [44], which modulates motor neuron activity, and *in vivo* testing using the Hoffman´s and blink reflexes activity on electromyography could detect subtle changes due to the effects of GlyR antibodies on the inhibitory pathways of the spinal cord [13, 14]. Active immunization models might provide a more sustained and aggressive immune response that could more faithfully reproduce the long‐term effects of GlyR‐Abs.

## Author contributions

The study was conceived by Angela Vincent with Alexander Carvajal‐González and Bethan Lang. Material preparation, data collection and analysis were performed by Alexander Carvajal‐González and Leslie Jacobson advised by Linda Clover and Bethan Lang and supervised by Angela Vincent. The plasmapheresis sample was provided by Mirdhu Wickremaratchi, and Simon Shields helped provide clinical information. The first draft of the manuscript was written by Alexander Carvajal‐Gonzalez, revised by Angela Vincent and all authors had the opportunity to comment on the manuscript and approve the final version.

## Conflicts of interest

AV and the University of Oxford hold patents and receive royalties and payments for antibody assays unrelated to the content of this manuscript. All other authors have no conflict of interest.

## Ethical approval

All procedures performed in the study were in accordance with the ethical standards of the Oxford University Regional Ethics Committee.

### Peer Review

The peer review history for this article is available at https://publons.com/publon/10.1111/nan.12666.

## Supporting information


**Data S1.** Supplementary methods.
**Figure S1.** Experimental protocol of systemic passive transfer of GlyR‐IgG into mice.
**Figure S2.** LPS produces temporary sickness and alters mouse behaviour.
**Figure S3.** PERM patient IgG transferred to mice was deposited in cerebellum but not hippocampus.

## Data Availability

The data that support the findings of this study are available from the corresponding author upon reasonable request.
