# Supplementary material for: Systemic delivery of human GlyR IgG antibody induces GlyR internalization into motor neurons of brainstem and spinal cord with motor dysfunction in mice
Source: Neuropathol Appl Neurobiol. 2020 Sep 28;47(2):316–27. doi: 10.1111/nan.12666 (PMC7873718; doi:10.1111/nan.12666)

Alexander Carvajal-González MD, MSc, PhD^1,2^, Leslie Jacobson PhD^1^, Linda Clover BSc^1^, MirdhuWickremaratchi^3^, Simon Shields^4^, Bethan Lang PhD^1^ and Angela Vincent MSc FMedSci FRS^1^

1. Nuffield Department of Clinical Neurosciences, John Radcliffe Hospital, Oxford OX3 9DU, UK.
2. Current address: Dr A Carvajal-González, Universidad El Bosque, School of Medicine, Bogotá, Colombia.
3. Hurstwood Park Neurological Centre, Brighton and Sussex University Hospitals NHS Trust , Lewes Road, West Sussex, RH16 4EX, UK
4. Neurosciences Department, Taunton and Somerset NS Foundation Trust, Musgrove Park Hospital, Taunton TA1 5DA, UK
5. **Supplementary Methods**

IgG Purification

Quantification of purified human IgG

Quantification of IgG in mice sera from passive transfer studies

Behavioural testing

Tissue preparation

1. **Supplementary Figures**

Fig.1. Experimental protocol of systemic passive transfer of GlyR-IgG into mice.

Fig.2. LPS produces temporary sickness and alters mouse behaviour.

Fig.3. PERM patient IgG transferred to mice was deposited in cerebellum but not hippocampus

1. **Supplementary Methods**

*IgG Purification (expanded version from main text)*

IgG fractions were purified from plasmapheresis filtrate of one patient obtained as part of standard clinical care from author (Simon Shields and Mirdhu Wickremaratch), and one healthy control adult male, using ammonium sulphate. The patient is described briefly in the main text. Ammonium sulphate was added to the samples until it reached a 45% saturation, the mix was left rotating overnight at 4°C and centrifuged (5000 G, 30 min) to remove the supernatant. The pellet was diluted in Ringer´s lactate solution (Baxter, USA) and dialysed against phosphate-buffered saline PBS (1:10) three times at RT; the last dialysis was performed overnight at 4°C. The following day, the IgG fractions were dialysed (1:10) two times in Ringer´s lactate (Baxter, USA), aliquotted, filter sterilized with 0.20 μm membranes and stored at -20°C until use.

*Quantification of purified human IgG*

Purified IgG fractions (diluted 1:300 in DNase free H2O) were mixed with appropriate amounts of NuPAGE sample reducing agent (10x) and LDS sample buffer (4x) (Invitrogen, CA, USA) and the mixed solution was boiled for 5 minutes at 95° C. 10 μl of the mixed sera and 5 μl of SeeBlue Plus2 pre-stained standard molecular weight marker (Invitrogen, CA, USA) were loaded onto 3-8% NuPAGE Bis-Tris SDS polyacrylamide gel lanes (Invitrogen, UK) and electrophoresed (150 mV for 1-1.5 hours) on a 4-12% bis-tris gradient gel and transferred using X Cell II BLOT (Invitrogen, UK) (25 mV for 30 min, 50 mV for 30 min) onto nitrocellulose membranes.

The nitrocellulose blots were blocked with 3% milk (Marvel) in PBS/1% tween for 1 hour and, to assess the success of the transfer, nitrocellulose membranes were rinsed with distilled water and reversibly stained with Ponceau S (0.1% (w/v) ponceau in 5% acetic acid) and scanned between two sheets of acetate film. Membrane blots were then incubated with rabbit anti-human IgG HRP (1:2000) for two hours at RT. The blots were washed three times with PBS/1% tween and developed with 3, 3’ diaminobenzidine (DAB-HCl, 50 mg/ml in PBS). Dried blots were then scanned and the 50 kD IgG heavy chain analysed with ImageJ (NIH, USA). IgG standards were run in parallel and the IgG concentrations from the purified fractions were calculated by comparison with standard IgG.

*Quantification of IgG in mice sera from passive transfer experiments*

To quantify the concentration of human IgG present in the mouse sera, animals were bled at day 14 during the perfusion procedure. Serum was obtained after blood centrifugation (13000 RPM, RT for 30 min). Mouse sera and purified human IgG were diluted to 1:100 and run on western blots as above.

*Time course of effect of intraperitoneal injection of LPS on mouse behaviour.*

LPS causes physiological and behavioural alterations due to the systemic release of pro-inflammatory cytokines. To see the duration of such effects, which could confound the results of the behavioural testing, 12 C57/BL6j male mice (6–8 weeks old, were randomized to receive LPS (3 mg/kg) (3 mice), saline (6 mice) or no-injection (3 mice). Measures of general health, temperature, motor behaviour, anxiety-like behaviour and weight were measured immediately after the injection of LPS and subsequently at 2, 6, 24 and 48 hours.

*Behavioural testing (expanded version from main text)*

Behavioural tests were selected with the aim to identify mouse behaviour that could be related to motor and neuropsychiatry manifestations observed in PERM patients. Before all the tests, the animals were habituated for 5 min to each instrument and to the room where the test took place in order to diminish the arousal and anxiety levels. Between mice, the walls and floor of the different instruments were cleansed with a moist tissue, followed by a dry tissue.

For motor evaluation, the following tests were used (Deacon, 2013):

1. Rotarod: The rod is 3 cm in diameter, supported 30 cm above the base of the apparatus with a knurled surface in a series of parallel ridges along the longitudinal axis, to enable mice grip. The rotarod is used for testing balance and coordination in rodents, using a continuous acceleration protocol. The start speed was adjusted to 4 rpm, the acceleration rate to 20 rpm/min. Maximum speed was 40 rpm. The variable analysed was time to fall, which was the average of five trials.
2. Grip strength: The inverted screen is a 43 cm square of wire mesh consisting of 12 mm squares of 1 mm diameter wire, surrounded by a 4 cm deep wooden beading to prevent the mouse attempts to climb on to the other side. The inverted screen evaluates the muscular strength in all four limbs in rodents, after placing the mouse in the center of the wire mesh screen, rotated to an inverted position and elevated 40-50 cm above a padded surface. The variable analysed was time to fall, which was the average of five trials.
3. Beam walking: The beam walking evaluates sensorimotor coordination and balance in rodents. A wooden rod 60 cm long and 8 mm wide was used, positioned 50 cms above the floor, supported by two pillars, with a box in the goal. The variables analysed were total time to cross, time to turn around, number of steps needed to cross and the number of errors (footfalls from the runaway).
4. Footprint analysis: Footprints obtained when rodents cross a wide runaway (7 cm wide and 60 cm long, covered with a strip of paper, positioned 50 cms above the floor and supported by two pillars, with a box in the goal). The variables analysed were total time to cross, number of steps needed to cross, number of errors (footfalls from the runaway) and stride length (left, right) and base support measurement changes.
5. Sensorimotor function: Walking over the narrow beam was qualitatively analysed using a slightly modified rating scale consisting of four distinct categories: coordinated stepping, tail use, postural control and footfall recovery. Each category was rated on a 3-point scale with a score of zero for absent movement, a score of one for atypical movements, and a score of two for typical movements (Metz and Whishaw, 2009; Shriner et al., 2009).

For neuropsychiatry evaluation, tests focusing on anxiety responses were used, the mouse behaviour was observed for 5 min and analysed as described (Deacon, 2013; Lezak 2017):

1. Light-Dark box: The dark and light box consisted of an open white compartment 30 x 20 x 20 cm joined by a 3 x 3 cm opening to a dark box 15 x 20 x 20 cm covered by a lid. The white compartment was illuminated by a 60 W anglepoise lamp placed 45 cm above the centre of the floor of the light side of the box. One side of the box was transparent, which permitted the observation of the mouse from the side after being placed in the centre of the light box facing away from the opening. The variables analysed were latency to enter the light compartment, time spent in the light and dark compartments, number of crosses between light and dark compartments.
2. Successive alleys: The apparatus consisted of four successive linearly connected alleys of putatively increasing anxiogenic character. Each alley was 25 cm long. Alley 1 had 25 cm high walls, was 8.5 cm wide and was painted in black. A 0.5 cm step down led to Alley 2 which was 8.5 cm wide, had 1.3 cm high walls and was painted grey. A 1 cm step down led to Alley 3 which was 3.5 cm wide had 0.8 cm high walls and was painted white. A 0.4 cm step down led to Alley 4 which was 1.2 cm wide, had 0.2 cm high walls and was painted white. The apparatus was elevated 50 cm from the floor and 10 cms of Alley 1 and all the others alleys were away from the stand. A mouse was placed at the closed end of alley 1 facing the wall. The variables analysed were latency to exit the closed alley, time spent in the closed alley and open alleys, number of crosses between the closed and the open alleys.
3. Open field: The open field was a dark enclosed arena of 50 x 50 cm divided into 10 cm squares illuminated with a 60 W anglepoise lamp placed 45 cm above the centre of the floor of the box. The open field also evaluates general motor activity, the mouse was placed into a corner square facing the corner. The variables analysed were number of peripheral crosses, number of central crosses, number of total crosses, number of rears.

All these data were recorded on spread-sheets and the main results presented in Table 2 in the main text.

Stiffness was rated semi-quantitatively, in the home cages and during the tests, similar to the clinical testing performed in humans with SPS (Dalakas et al., 2000). Scores are on a scale of 0 to 4: 0. Absent. 1. Slowing when in free motion; 2. Trunk stiffness during walking; 3. Reduced climbing ability and 4. Muscle spasms and dystonic movements. None of these were observed in the home cages and only on the rotarod was there evidence of reduced walking ability.

*Tissue preparation (extended version from the main text)*

At the end of the experiments, mice were deeply anesthetized with intraperitoneal injections of ketamine and xylazine (100 mg/kg and 5 mg/kg, respectively) and perfusion surgery was performed on mice. Then while the heart was still beating, an intracardiac catheterization with a needle in the apex of the left ventricle was performed and blood was withdrawn from the heart with a transcardiac perfusion of 200 to 300 cc of phosphate buffered saline (PBS). The whole brain and spinal cord were removed and dropped into pre-cooled isopentane (2-methylbutane) on dry ice to snap-freeze the tissue, the spinal cord was embedded in optimal cutting temperature compound (OCT compound) (Tissue Tek, Germany) for tissue stability during cutting. Frozen brains and spinal cords were wrapped with aluminium foil, transferred into a plastic vial and stored at -80°C until needed for cutting.

Fresh frozen sections without paraffin embedding of the whole brain and spinal cords were placed at the cryostat (Leica 1850 CM, Germany) and allowed to reach cutting temperature at -20°C to avoid shattering. Eleven-micron thick cryostat sagittal frozen sections of the whole mouse brain and coronal sections of the spinal cord were thaw-mounted on SuperFrost Plus glass slides and stored at -20ºC until needed for immunohistochemistry which is described in the main text.

1. **Supplementary figures**

**Supplementary Figure 1. Experimental protocol of systemic passive transfer of GlyR-IgG into mice.**

***Effects of LPS:*** Mice were injected ip with LPS (3 mg/kg n=3) or saline (n=6), or not injected (n=3). Behavioural testing was performed before the injections and for 48 hours after LPS injection.

***Effects of GlyR-IgG antibodies:*** GlyR-IgG (12.4 mg/ml, n=6) or the healthy control purified IgG (control IgG, 14.6 mg/ml, n=6) were administered ip daily over 12 days (0.5 ml), and one group was given saline injections (n=6) (c); all three were given LPS ip on days 3 and 8 (3 mg/kg and 1.5 mg/kg respectively). A fourth group was left un-injected (n=6). Behavioural testing was done twice at baseline and after the LPS injections (days 5,6,7 and 10,11,12). At the end of the experiment, the animals were euthanized; blood was collected for serum testing and the brains processed for immunopathology. Abbreviations: LPS: Lipopolysaccharide; IP: Intraperitoneal; D: Day; IgG: Immunoglobulin.

**Supplementary Figure 2. LPS produces temporary sickness and alters mouse behaviour.** **a.** LPS treated mice had a significant decrease in temperature reversed by 48 hrs. Saline and un-injected mice showed no change over time (Two-way ANOVA F (2, 45) = 36.24; p < 0.0001). **b.** LPS treated mice had a progressive decrease in weight that was significant after 48 hrs compared with the two non-LPS mice (Two-way ANOVA F (4, 45) = 34.91; p = 0.0052). **c.** LPS treated mice had a larger decrease in the percentage of peripheral crosses compared to that in saline and un-injected mice, but this reversed by 48 hrs. **d.** LPS treated mice required a higher number of steps to cross the narrow beam, but this also reversed by 48 hrs (Two-way ANOVA F (2, 36) = 25.14; p < 0.0001). **e.** LPS treated mice required a higher number of steps to cross the wide walkway, but this reversed by 24 hrs (Two-way ANOVA (F (2, 36) = 5.033; p = 0.0118). **f.** The LPS and the saline groups showed marked reduction in the numbers of crosses between the light and dark boxes over time, compared to the un-injected mice, but the results at baseline were highly variable and the final results were not different; it is likely that these changes were influenced by habituation to the test (Two-way ANOVA (F (2, 36) = 1.095; p = 0.3455).

**Supplementary Figure 3.** **IgG transferred to mice co-localised with GlyRs in the cerebellum (a) but not in the hippocampus (b).** In the cerebellum of injected mice, human IgG (green) colocalised with von Willebrand factor and also with GlyRs, only in mice injected with GlyR-Abs and not those injected with HC IgG. In the hippocampus, there was very little GlyR expressed or human IgG in either the GlyR-Ab injected or HC-injected brains. Nuclei are stained with DAPI (blue). Poly-Ab2 = polyclonal antibody to GlyRα1. Double-labelled photographs were taken at 40x magnification.

**References (also in main text)**

1. Deacon RM. Measuring motor coordination in mice. J Vis Exp 2013; (75): e2609.
2. Deacon RM. Measuring the strength of mice. J Vis Exp 2013; 2: (76).
3. Deacon RM The successive alleys test of anxiety in mice and rats. J Vis Exp 2013; (76): 2705.
4. Lezak KR, Missig G, Carlezon WA Jr. Behavioral methods to study anxiety in rodents. Dialogues Clin Neurosci 2017; 19(2): 181-191.
5. Metz, G. A. and I. Q. Whishaw. The ladder rung walking task: a scoring system and its practical application. J Vis Exp 2009; (28).
6. Shriner, A. M., F. R. Drever, et al. The development of skilled walking in the rat. Behav Brain Res 2009; 205(2): 426-435.
7. Dalakas MC, Fujii M, Li M, McElroy B. The clinical spectrum of anti-GAD antibody-positive patients with stiff-person syndrome. Neurology 2000; 55: 1531-5.


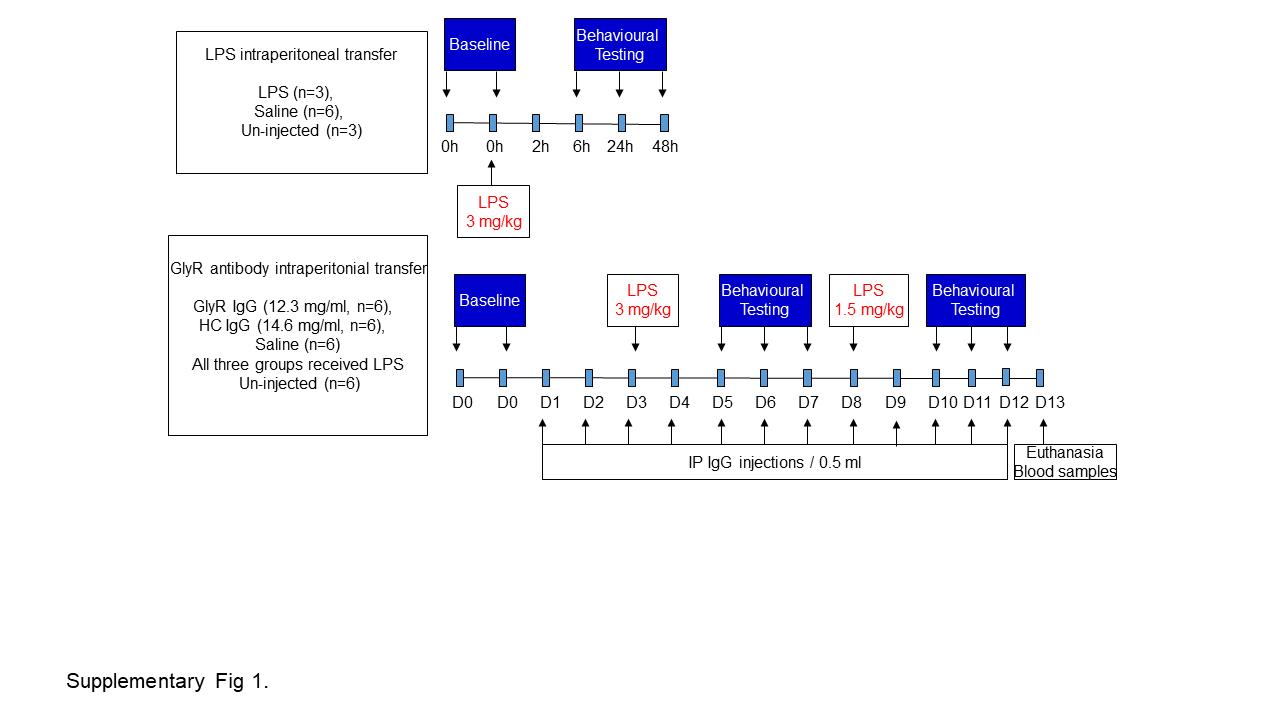

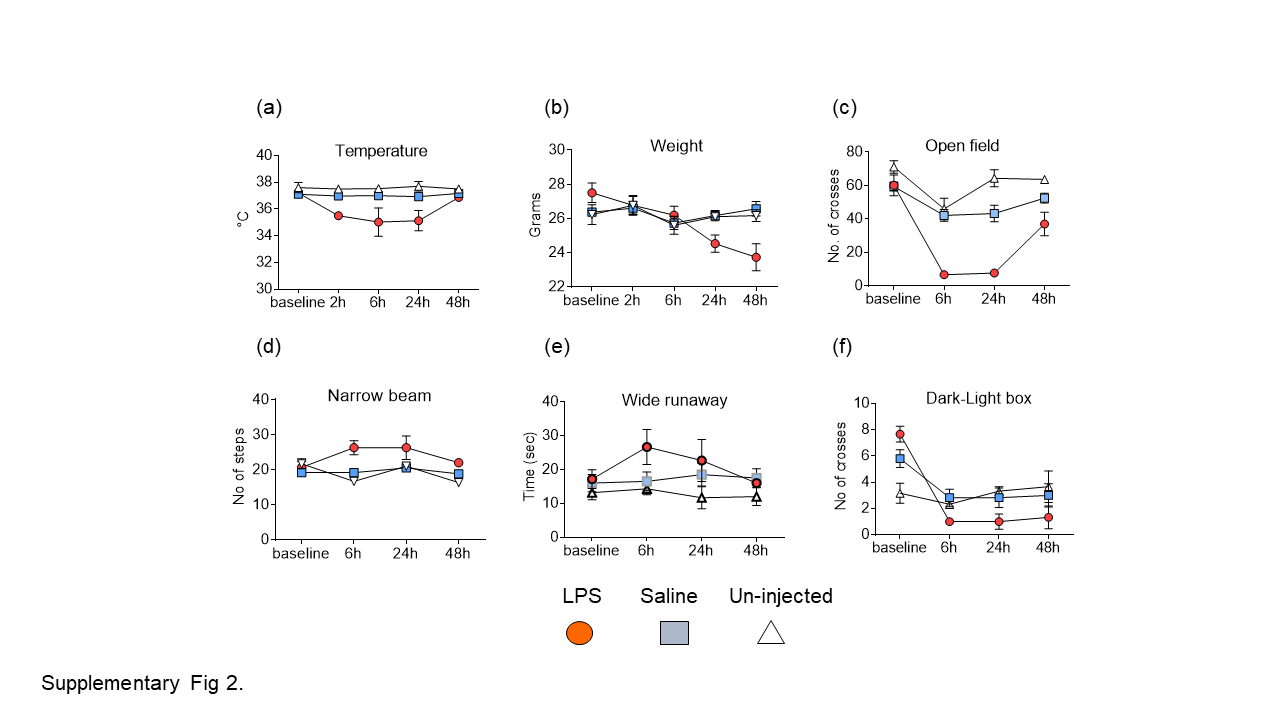

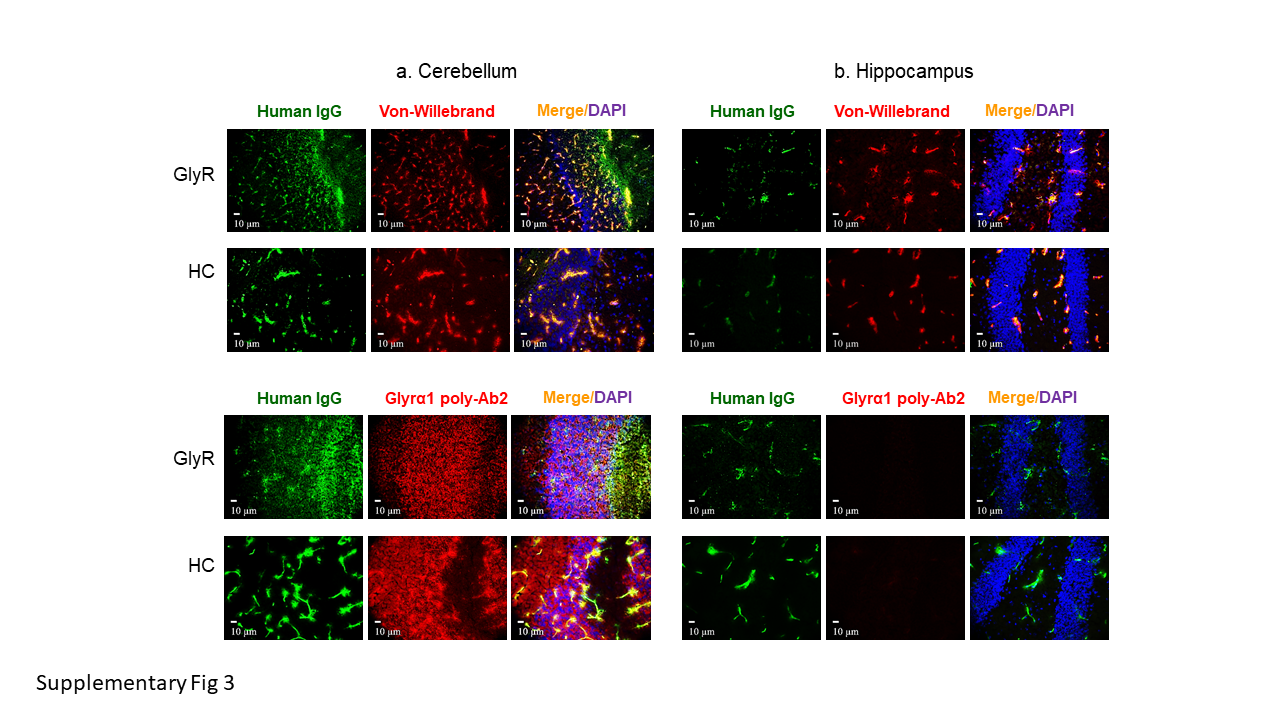

Supplement: Supplementary file 1 — Data S1. Supplementary methods. Figure S1. Experimental protocol of systemic passive transfer of GlyR‐IgG into mice. Figure S2. LPS produces temporary sickness and alters mouse behaviour. Figure S3. PERM patient IgG transferred to mice was deposited in cerebellum but not hippocampus. [file NAN-47-316-s001.docx]
